# ASO Author Reflections: Virtual Multimodal (P)rehabilitation for Cancer Surgery: Hope or Hype?

**DOI:** 10.1245/s10434-026-19465-9

**Published:** 2026-03-26

**Authors:** Jack Reeves, Cherry Koh, Michael Solomon, PRIORITY-CONNECT Collaborators, Allan Ben Smith, Amy Cao, Andrew Sutherland, Angus Lee, Annie Zhou, Aveline Chan, Basil D’Souza, Bernhard Riedel, Briana Shailer, Carlo Pulitano, Charbel Sandroussi, Cheryl Tobler, Chris Gillespie, Christopher Byrne, Claire Jeon, Danette Bianca Wright, David Clark, Dayan De Fontgalland, Derek Cunningham, Deshitha Gardiye Hewawasam Thuduwage, Douglas Stupart, Fernando Arduini, Freya Rubie, Gaynor Beardsworth, Gino Iori, Helen Mohan, Haryana M. Dhillon, Howard Tang, Jacob McCormick, Jerome Laurence, Jessica Connell, Joseph Cherng Huei Kong, Ju Young Cheong, Justing Yeun, Kate White, Kate Wilson, Kathryn Cherry, Kim Delbaere, Kimberley Bostock, Kirk Austin, Kym Sheehan, Lauren Reece, Leanne Hassett, Lilian Whitehead, Liliana Laranjo, Linda Denehy, Margaret Allman-Farinelli, Manpreet Aulakh, Marine Salter, Mbathio Dieng, Michael Suen, Nabila Ansari, Nicholas Hirst, Nima Ahmadi, Olivia Dwyer, Olivia Martin, Owen Hutchings, Peter Lee, Phyllis Butow, Qiang Li, Rachael L. Morton, Rajni Lal, Rihan Shahab, Rika Johnander, Robert Winn, Sharon Carey, Stephen Jancewicz, Stephen Smith, Tarik Sammour, Thomas Poulton, Toan Pham, Vicki Patton, Vinna An, Xiaoqiu Liu, Kate Alexander, Sascha Karunaratne, Daniel Steffens

**Affiliations:** 1 Surgical Outcomes Research Centre (SOuRCe), Royal Prince Alfred Hospital, Sydney, NSW Australia; 2 Discipline of Physiotherapy, Graduate School of Health, Faculty of Health, University of Technology Sydney, Sydney, NSW Australia; 3 Physiotherapy Department, Royal Prince Alfred Hospital, Sydney, NSW Australia; 4 NHMRC Clinical Trials Centre, Faculty of Medicine and Health, The University of Sydney, Sydney, NSW Australia; 5 Institute of Academic Surgery (IAS), Royal Prince Alfred Hospital, Sydney, NSW Australia

## Past

Postoperative complications and adverse outcomes remain a significant burden after gastrointestinal cancer surgery, affecting patient recovery, quality of life, and healthcare resource utilization.^1,2^ Although (p)rehabilitation (combining preoperative and postoperative rehabilitation) has attracted growing interest as an intervention to improve surgical outcomes, the current evidence base is largely derived from face-to-face delivery models. Such models present considerable challenges for equitable healthcare access, particularly across geographically dispersed populations such as in Australia. Virtual delivery of (p)rehabilitation offers a scalable, patient-centered solution that can be integrated early into the preoperative care pathway, reducing barriers related to distance and mobility.

## Present

We aimed to determine whether a virtual multimodal (p)rehabilitation program, delivered up to 6 weeks preoperatively and 3 months postoperatively, was feasible and acceptable for patients undergoing gastrointestinal cancer surgery. The multimodal hub provided participants with specialized multidisciplinary input from a physiotherapist, psychologist, dietitian, specialist nurse, social worker, and geriatrician, targeting modifiable risk factors preoperatively (including physical deconditioning and malnutrition), preparing participants for surgery through expectation setting, and minimizing physiological and psychological decline associated with neoadjuvant treatments and/or surgery. Our pilot trial^3^ confirmed that this virtual delivery model was both feasible and acceptable, with strong uptake (65%), retention (95%), and adherence (74%) and high overall participant satisfaction.

## Future

Building on these promising findings, PRIORITY-CONNECT 2 has advanced to a full-scale phase III multicenter hybrid type 1 effectiveness-implementation randomized controlled trial. The trial will recruit 564 participants across multiple Australian sites to determine the effectiveness of the virtual multimodal hub in reducing postoperative complications after colorectal cancer surgery. Funded by the Medical Research Future Fund, PRIORITY-CONNECT 2 represents Australia's largest (p)rehabilitation trial to date, with findings expected to inform national and international clinical practice guidelines and shape the future delivery of perioperative cancer care.
